# Recall of pain and discomfort during oral procedures experienced by intubated critically ill patients in the intensive care unit: A qualitative elicitation study

**DOI:** 10.1080/24740527.2020.1732809

**Published:** 2020-09-24

**Authors:** Craig M. Dale, Sarah Carbone, Alicia Lara Gonzalez, Karen Nguyen, Julie Moore, Louise Rose

**Affiliations:** a Lawrence S. Bloomberg Faculty of Nursing, University of Toronto, Toronto, Ontario, Canada; b Department of Critical Care, Humber River Regional Hospital, Toronto, Ontario, Canada; c Department of Medicine, Sunnybrook Health Sciences Centre, Toronto, Ontario, Canada; d Department of Critical Care, Mount Sinai Hospital, Toronto, Ontario, Canada; e Florence Nightingale Faculty of Nursing, Midwifery and Palliative Care, King’s College London, London, UK; f Department of Critical Care Medicine and Sunnybrook Research Institute, Sunnybrook Health Sciences Centre, Toronto, ON, Canada

**Keywords:** elicitation, intensive care units, mechanical ventilation, oral care, oral health, patient-oriented research, procedural pain, qualitative research

## Abstract

**Background**: Intubated and mechanically ventilated patients in the intensive care unit (ICU) may experience pain during routine oral procedures such as oral suctioning and tooth brushing. Despite the importance of pain prevention and management, little is known about patients’ experiences of procedural oral pain.

**Aims**: The aim of this study was to explore patients’ recollections and recommendations for pain and discomfort during routine oral procedures.

**Methods**: A qualitative descriptive design was used. Adult patients were recruited from a mixed medical–surgical–trauma ICU in an academic hospital in Toronto, Canada. Participants were interviewed using object elicitation methods within 7 days of discharge from the ICU. Data were analyzed using directed content analysis methods.

**Results**: We recruited 33 participants who were primarily male (23, 70%), with an average age of 54 (SD = 18) years, admitted with a medical (13, 39%), trauma (11, 33%), or surgical (9, 27%) diagnosis and dentate (27, 82%). Most participants described oral procedures as painful, discomforting, and emotionally distressing. Identified sources of pain included dry, inflamed oral tissues and procedural technique. Procedural pain behaviors were perceived to be frequently misinterpreted by clinicians as agitation, with consequences including physical restraint and unrelieved suffering. Participants advocated for greater frequency of oral care to prevent oral health deterioration, anticipatory procedural guidance, and structured pain assessment to mitigate the dehumanizing experience of unmanaged pain.

**Conclusions**: Patients described routine oral care procedures as painful and recalled suboptimal management of such pain. Procedural oral pain is an important target for practice improvement.

## RÉSUMÉ

**Contexte**: Les patients intubés et ventilés mécaniquement dans l'unité de soins intensifs (USI) peuvent ressentir de la douleur lors des procédures buccales de routine telles que l'aspiration et le brossage de dents. Malgré l'importance de la prévention et de la prise en charge de la douleur, on sait peu de choses sur l’expérience des patients en matière de douleur buccale liée aux procédures.

**Objectifs**: Cette étude a pour but d'étudier les souvenirs et les recommandations des patients concernant la douleur et l'inconfort lors des procédures buccales de routine.

**Méthodes**: Un modèle descriptif qualitatif a été utilisé. Les patients adultes ont été recrutés dans une unité mixte de soins intensifs médico-chirurgicaux et de traumatologie d'un hôpital universitaire de Toronto, au Canada. Les participants ont été interrogés à l'aide de méthodes d’élicitation des objets dans les sept jours suivant leur sortie de l'unité de soins intensifs. Les données ont été analysées à l'aide de méthodes d'analyse de contenu dirigée.

**Résultats**: Nous avons recruté 33 participants qui étaient principalement des hommes (23, 70 %), âgés de 54 ans (É.-T. 18), admis avec un diagnostic médical (13,3 9 %), traumatique (11, 33 %) ou chirurgical (9, 27 %) et dentés (27, 82 %). La plupart des participants ont décrit les procédures orales comme étant douloureuses, inconfortables et émotionnellement pénibles. Les sources de douleur répertoriées comprenaient les tissus buccaux secs et enflammés et la technique d'intervention. Les comportements douloureux liés aux procédures ont été perçus comme étant souvent interprétés à tort par les cliniciens comme de l'agitation, avec pour conséquences une contrainte physique et une souffrance non soulagée. Les participants ont plaidé pour une plus grande fréquence des soins bucco-dentaires afin de prévenir la détérioration de la santé bucco-dentaire, pour des conseils procéduraux anticipés et pour une évaluation structurée de la douleur afin d'atténuer l'expérience déshumanisante de la douleur non prise en charge.

**Conclusions**: Les patients ont décrit les soins bucco-dentaires de routine comme étant douloureuses et se sont souvenus de la prise en charge sous-optimale de cette douleur. La douleur buccale procédurale est un objectif important pour l'amélioration de la pratique.

## Introduction

Critically ill adults admitted to the intensive care unit (ICU) experience pain during routine therapeutic procedures.^1^ For example, those receiving invasive mechanical ventilation report moderate to severe pain during physical repositioning, wound care, and drain removal.^2^ Psychological symptoms such as anxiety resulting from the inability to self-report pain due to intubation are known to magnify pain.^3^ Short-term outcomes of unrelieved pain include sleep impairment, immunodepression, infection, and delirium.^4^ Long-term outcomes of pain include cognitive and functional impairment^5^ and development of chronic pain.^6^ Poor pain outcomes support a foundation for the rigorous assessment and prevention of procedural pain in ICU patients.^7^

Recent developments in pain inquiry include examination of procedures involving the mouth and throat, such as oral suctioning of secretions and preventative oral hygiene.^8^ A constellation of dehydrating oral stressors during ICU treatment contribute to reduced salivary flow and xerostomia (i.e., severe dryness).^9^ Loss of buffering, lubricating, and antimicrobial properties of saliva result in reduced oral pH, inflammation, and infection.^10,11^ Ensuing oral diseases such as mucositis (inflammation, ulceration and hemorrhage of mucosal tissues), candidiasis (yeast/fungal infection of the mucous membranes), odynophagia (painful swallowing), gingivitis (gum inflammation), and tube-related (e.g., endotracheal, orogastric) pressure injury may render procedures with toothbrushes, swabs, antimicrobial solutions, and suction devices painful. As global demand for ICU treatment is expected to increase,^12^ oral procedural pain is an important topic for knowledge development.^13^

Failure to assess and manage procedural pain may result from inadequate knowledge of the pain experienced by patients and/or communication difficulties between the patient and health care team.^4,14^ Procedural pain management is an important patient-identified target for ICU practice improvement.^5,15^ However, studies exploring patient recollections and recommendation for oral pain are notably lacking. A recent paradigm shift to light sedation during mechanical ventilation suggests more patients will be aware of pain but unable to self-report.^16,17^ Patients’ recollections of care processes provide invaluable insights for improvements in the quality of care.^18^ To the best of our knowledge, no study has explored intubated and mechanically ventilated patient recollections of and recommendations for pain during routine oral procedures.

## Methods

### Objectives

This qualitative study was embedded in a larger research project studying the prevalence of adult ICU patient behaviors during oral care.^8^ In this article, we report on the procedural oral pain recollections of intubated and mechanically ventilated patients, what it is like to experience such pain, and their recommendations for practice improvement.

### Research Design

A qualitative description (QD) design was employed in order to better understanding experiences of routine oral care processes.^19^ In QD, participants are asked to describe an experience in their own words and to suggest ways to improve those experiences or outcomes.^20^ A tenet of QD is analysis and reporting of events in concrete terms; it prioritizes participants’ verbatim accounts over researchers’ interpretations.^21^ Qualitative inquiry acknowledges how technical or scientific priorities of care can overshadow the human dimension of illness and its treatment. In positioning participant experiences as essential evidence, QD offers a special opportunity to address neglected dimensions of medical treatment, including procedural pain.

### Setting and Sample

Participants were prospectively recruited from a cohort of patients observed in the parent study following discharge from a mixed medical–surgical–trauma ICU in a university-affiliated hospital in Toronto, Canada, from September 2016 through January 2017. As a level 3 ICU, the study unit is provincially designated to provide invasive mechanical ventilation and life support for adult patients experiencing multisystem organ failure. The unit follows recommendations for analgosedation, which involves treating pain first and providing sedatives only when necessary to facilitate rest and reduce anxiety.^22^ Ventilated patients were nursed using a 1:1 ratio and received a unit-based oral hygiene regimen including toothbrushing every 12 h, suctioning of oral secretions every 1 to 4 h, and topical oral application of an antimicrobial solution (chlorhexidine gluconate 0.12%) using a swab every 6 h.

We anticipated that 25 to 30 patients would be required to qualitatively establish patterns of experience suitable for the development of themes.^23^ Eligible patients (1) were 18 years of age or older; (2) were orally intubated in the ICU for 48 h or longer; (3) recollected having an endotracheal tube; (4) were competent to provide informed consent; (5) were able to verbalize or communicate by other means; and (6) were available to participate in an interview within 7 days of ICU discharge. Patient exclusions were (1) inability to recall the endotracheal tube; (2) unable to communicate in English; and (3) experienced a condition or treatment not permitting routine oral care during the ICU encounter.

### Ethics

The participating hospital and University of Toronto research ethics boards approved the study (132-2015). Eligible participants were approached by a research assistant following ICU discharge to introduce the study and appraise their interest in participating. Written informed consent was obtained from each participant prior to study enrollment.

### Data Collection

Consenting patients participated in one 60-min in-person interview conducted by the lead investigator. Each interview took place at the patient’s hospital bedside within 7 days of ICU discharge using a semistructured interview guide. Because pain can be difficult to characterize in words, we employed an approach called object elicitation (i.e., objects paired with in-depth interviews).^24^ We presented participants with oral devices used in the study ICU (e.g., endotracheal tube, Yankauer oral suction device, toothbrush, and sponge-tipped swab) to trigger memory and tacit insights into oral care procedures. Elicitation is recommended to facilitate recall and presentation of new or unexpected aspects of an experience and minimize socially preferred responses to interview questions.^25–27^ We invited participants to rate their highest recollected procedural pain score on a 0 to 10 numeric rating scale, with 0 being *no pain* and 10 being *the worst pain possible*.^28^ Interviews were digitally recorded and transcribed verbatim. Patient demographics and treatment characteristics were collected from the medical record.

### Data Analysis

We used content analysis as our primary data analysis method.^29^ Content analysis is a method for describing the content of communication in a concise and systematic manner. Following independent reading of the transcripts, four researchers (CD, SC, LG, KN) met to discuss preliminary codes and build a codebook.^30^ To enhance our understanding of participant experiences, we conducted a manifest content analysis of pain and discomfort descriptors.^31^ As a final step, we organized coded narratives across four inductively derived thematic pain categories: descriptors of pain and emotional distress in participants’ own words, perceived sources or contributors to pain, examples of unrecognized suffering, and recommendations for professional care and support. NVivo10 software was used for coding and organization of data.^32^ For reporting numeric pain intensity data and patient characteristics, we used means and standard deviations or medians for continuous variables and frequencies and percentages for categorical variables.

## Results

We recruited 33 patient participants who were primarily male (23, 70%), with an average age of 54 (SD = 18) years, admitted with a medical (13, 39%), trauma (11, 33%), or surgical (9, 27%) diagnosis and dentate (27, 82%). All patients (33, 100%) received mechanical ventilation through an oral endotracheal tube; 15 (45%) had additional indwelling oral devices. The mean ICU length of stay was 13 (SD = 9) days. Participants reported a significant oral symptom burden during intubation, with procedural oral pain (17, 51%), generalized oral discomfort (29, 88%), and oral dryness (31, 94%) predominating. The mean ICU procedural oral pain intensity score was 7 (range 4–8). Oral pain (11, 33%), generalized discomfort (20, 60%), and dryness (26, 79%) persisted in the 7 days following ICU discharge. Other oral problems reported in the post-ICU period included changes to voice quality (23, 70%) and dysphagia (12, 36%; Table 1).

**Table 1.** Demographic characteristics.

*n* = 33	*n* (%)
Age, years, mean (SD), range	54 (18), 21–81
Sex, male	23 (70)
Dentate	27 (82)
Edentate	6 (18)
ICU admission category	
Medical	13 (39)
Surgical	9 (27)
Trauma	11 (33)
ICU length of stay, mean (SD), range	13 (9), 3–47
Ventilator mode	
Controlled	11 (33)
Spontaneous	22 (67)
Physical restraint	20 (60)
ICU oral devices	
Endotracheal tube	33 (100)
Orogastric tube	11 (33)
Bite block	2 (6)
Post-pyloric tube	2 (6)
Duration of intubation, mean (SD), range	8 (4), 2–16
ICU oral symptoms	
Procedural pain	17 (51)
Generalized discomfort	29 (88)
Dryness	31 (94)
Gagging	21 (64)
Dysgeusia	6 (18)
ICU procedural oral pain score, mean (SD), range	7 (1.5), 4–8
Post-ICU oral symptoms	
Oral pain	11 (33)
Generalized discomfort	20 (60)
Dryness	26 (79)
Voice changes	23 (70)
Dysphagia	12 (36)
Post-ICU oral pain score, mean (SD), range	2 (1.3), 0–4
Dental insurance	16 (48)

ICU = intensive care unit.

The following sections are organized by thematic pain categories, including descriptors of pain and emotional distress, sources of pain, unrecognized suffering, and professional care and support. The main findings indicate that pain and suffering remain an important issue for patients despite clinical emphasis on analgosedation and patient-centered care.

### Descriptors of Pain and Emotional Distress

Table 2 displays common descriptors employed by patients to explain oral pain at rest and during routine oral procedures. Almost all participants used the terms *pain* and *discomfort* to describe their oral health state and the experience of routine oral procedures during mechanical ventilation:

The pain was in the throat and in the back of the tongue. (P14)

It was just 24/7 discomfort. The [mouth] dryness makes it a lot harder to breathe. It affects your breathing, maybe not harder, but it makes it sort of painful everywhere. And the other part is you start to get raw from it. So everything is starting to sting. (P2)

**Table 2.** Oral pain descriptors.

Pain descriptors	Descriptor frequency (*n*)	Weighted frequency (%)^a^
Pain	319	0.90
Discomfort	109	0.31
Bad	72	0.20
Sore	60	0.17
Hurt	48	0.13
Unpleasant	38	0.11
Terrible	28	0.08
Choking	23	0.06
Fear	20	0.06
Bothersome	19	0.05
Horrible	13	0.04
Tight	12	0.03
Rough	11	0.03
Constant	10	0.03
Drill	8	0.02
Irritation	6	0.02
Scratch	5	0.01
Tender	4	0.01
Sting	4	0.01
Fearful	3	0.01
Sharp	3	0.01
Numb	3	0.01
Burning	3	0.01
Panic	3	0.01

^a^Weighted Percentage is the frequency of the word relative to the total words counted.

Some described pain as a constant feeling of tightness or choking in the throat. The introduction of hygiene instruments and antimicrobial solutions into the oral space, concurrent to the feeling of tightness, was characterized as “hurtful,” “unpleasant,” and even “horrible”:

You don’t want them to do it. It hurt. (P28)

Most patients described pain and discomfort as evoking strong emotions such as fear, anguish, and worry. Several participants employed metaphors when attempting to communicate the distressing sensation of the Yankauer suction device being used to remove oral secretions with vacuum pressure:

I remember it felt like bones. I found it really uncomfortable. (P22)

Very painful. Like crushed nails. (P12)

In contrast to the disturbing and threatening sensation associated with oral suctioning with a hard plastic device, most patients described the sponge swab as a “comforting” device in the mouth:

Oh yeah. Those green swabs, I became good friends with those. (P32)

Participants explained how comfort was derived from care provided with sponge swabs, possibly due to their soft, pliable nature and capacity to transport much desired “moisture” to dry tissues and a pleasant “minty flavor” from mouth rinses.

### Sources of Pain

A prominently reported antecedent of procedural oral pain was oral health deterioration following endotracheal intubation. Discomforting oral health changes were perceived to result from dehydrating stressors such as a continuously open mouth and injury from orally placed devices. Two participants explained how these circumstances led to discomfort when opening the mouth to accommodate instruments for oral procedures:

It was so dry that the corners [of my lips] cracked and it hurt to move my mouth. (P23)

The suction and the [swab] could evoke pain just because everything in there was very tender. (P25)

Some patients recollected a convergence of painful oral health problems including, but not limited to, severe dryness, weeping sores in the corners of the mouth, fungal infection, and mucosal pressure ulcers. These and other conditions, including preexisting dental health problems, contributed to reactivity during oral procedures, which could result in involuntary self-inflicted bite injuries:

I remember that it was super dry all the time. I guess at some point during the whole ICU process, I [bit down] real bad on this part of my mouth. It’s all raw in there. (P4)

Lack of time, skill, and attention on the part of clinicians was seen as an important contributor to procedural pain. For example, some clinicians were perceived to be overly “mechanical,” “rough,” or “rushed” when passing instruments inside the mouth, unavoidably stimulating sensitive tissues:

Sometimes the [nurse] might stick the swab in a bad spot. (P29)

When the suction was going right to the back and stuff like this. It hurt. (P7)

Elicitation of a gag and cough reflex, reported as a common response to rushed or unskilled oral procedures, was described as contributing to a cascade of pain throughout the body:

It is really painful with the broken ribs … gagging and choking the whole time. (P6)

Movement associated with gagging and coughing exacerbated bodily pain from admission-related orthopedic trauma (e.g., rib fractures), surgery (e.g., incisions/drains), and infected or inflamed tissues (e.g., cellulitis).

### Unrecognized Suffering

Several patients perceived clinician misunderstanding of their attempts to nonverbally communicate procedural discomfort. For example, a few participants recalled reaching with their hands to adjust oral and nasal tubes or to convey the need to slow down or stop an oral procedure:

Yeah, I remember because it was painful and discomforting, so I tried to reach there. (P22)

Others recollected wincing, grimacing, or biting down on the endotracheal tube, swabs, toothbrushes, and suction devices as a spontaneous response to procedural discomfort. Rather than recognizing and responding to their pain, however, clinicians warned the patient not to interfere with the devices in their mouth:

They just told me, “Don’t do that.” That’s all they kept telling me. (P9)

In response to these admonishments, participants felt vulnerable and scared. Four participants reported clear recollections of attempting to self-extubate when they could no longer tolerate the oral discomfort associated with the endotracheal tube or oral care procedures. One patient described prolonged discomfort leading to a state of “panic.” Similar to the experiences noted above, participants did not recollect appraisal of pain by the clinical team following attempted self-extubation. Instead, participants recalled that such events resulted in the application of bilateral wrist restraints that maintained the patients’ hands at their side and away from the mouth.

Participants reported persistent oral problems on the ward or step-down unit in the first 7 days following ICU discharge:

I feel like my teeth are coming out. I can move them with my tongue. (P15)

My teeth hurt, especially with hard foods. (P17)

Symptoms during this time included oral pain throughout hygienic care, generalized mouth and throat discomfort, and unremitting oral dryness. Most participants reported changes in voice quality following extubation, and several described oral discomfort contributing to difficulty eating and swallowing.

### Professional Care and Support

When asked whether there was anything that could have been done to decrease procedural pain and its consequences, participants recommended early and ongoing delivery of a comprehensive regimen of preventative oral care during mechanical ventilation. Variable delivery of oral care during the ICU stay informed an understanding of professional care for physical and psychological comfort:

Some of the nurses were very protective of me and they helped. They also were rather understanding of what to do and they made sure mouth care was done during the 12 hours that they were involved with me. However, mouth care is really scanty in the night shift. You know when the nurses and doctors take their breaks. For me that was a time I was so afraid to go to sleep. (P22)

Unmet oral health needs (i.e., severe dryness) were perceived to contribute to discomfort at rest, pain during procedures, and even sleeplessness. Therefore, maintenance of healthy gums, teeth, and mucosal tissues was considered fundamental to overall patient well-being. Recommended interventions to maintain oral health included regular toothbrushing, moisture application, anticipatory guidance as to what was going to happen, and assessment of pain and discomfort during oral procedures.

In response to the presentation of oral care tools during interviews, few patients reported recollection of toothbrushing to control dental plaque, prevent gingivitis, and stimulate production of saliva during mechanical ventilation:

I don’t recall ever somebody brushing my teeth. (P27)

I never saw this toothbrush. (P31)

Accordingly, lack of toothbrushing was seen as a modifiable care process with implications for the prevention of swollen, painful gums:

I think it would be a good idea to brush teeth, but you got to be kind of gentle. Some of them are a bit rough. Yeah, there was one nurse … it was almost like she had a toothbrush on the end of a drill. (P24)

Similarly, more frequent application of moisture to the lips and anterior mouth using water or a commercially available moisturizer was considered an indispensable strategy:

The moisture. It helped. It eased the discomfort a little. (P9)

[The swab] doesn’t hold much water, but it was a step in the right direction. (P19)

As a nonpharmacological pain management strategy, delivery of moisture via sponge swabs, ice chips, mouth rinses, and topical moisturizers was perceived to ameliorate symptoms of dryness and pain. Several participants endorsed preprocedural analgesia, when appropriate, to manage the discomfort of instruments on inflamed oral tissues and wounds. The Yankauer suction device was described as the most painful instrument used during oral procedures and one for which preprocedural analgesia should be considered.

Participants described poor verbal preparation for oral procedures. This left many feeling unprepared for or surprised by the placement of oral instruments and antimicrobial solutions inside the mouth. The lack of preparation was perceived to limit patients’ ability to remain calm and cooperate by holding their mouth open:

It would have been much more beneficial if they told you in advance and that way you can be prepared for what you’re about to get in your mouth. (P22)

Information would help out a lot. Every time they do it, somebody comes around and just sticks the tube in. As long as you know what’s going on … just thinking ahead, when it’s going to be over, and it’ll be better when it’s over. (P6)

Poor visibility of oral tools (e.g., toothbrush, swabs, Yankauer) placed inside the mouth further contributed to misapprehension about what was happening to them. As a modifiable approach to care, participants proposed anticipatory verbal preparation and step‐by‐step guidance as an essential component of patient‐centered oral procedures.

In speaking to expectations for quality and safety in health care, participants urged greater emphasis on the patient experience. For example, few participants recollected clinicians inquiring whether oral comfort was desired or achieved:

I think its one of the areas that has to be looked at for the overall well-being of the person. […] Even on the mental point of view, knowing that somebody’s thinking of you to say, “Hey, let’s give your teeth a little brush,” or “How about a little mouth wash?” to make you feel more like a human being. (P27)

Participants advocated for standardized procedural pain assessment and management to mitigate the potential for dehumanizing experiences, which were defined as treatment without pain and comfort being addressed.

In summary, key participant recommendations for procedural pain management included frequent oral hygiene, including toothbrushing, moisture application, anticipatory guidance, and pain appraisal and management.

## Discussion

To our knowledge, this is the first qualitative study to explore patient recall and recommendations for procedural oral pain and discomfort during invasive mechanical ventilation. Through interview and object elicitation, we investigated patients’ experiences of pain and thematically reported our results as descriptors of pain and emotional distress, sources of pain, unrecognized suffering, and professional care and support. The results of the present study suggest that ICU patients recollect unmet needs for oral pain management despite current emphasis on analgosedation.^7^ Participants described a complex relationship to oral care procedures; some interventions offered comfort, whereas others precipitated pain. Patient inability to self-report pain and distress posed consequences for physical and psychological safety. Misinterpretation of pain behaviors as agitation resulted in some patients experiencing physical restraint and unrelieved oral discomfort. Recommendations for procedural pain management included prevention of oral health deterioration through more frequent provisions of oral care, verbal guidance during oral procedures, and procedural pain appraisal.

Our results align with findings from recent video and photographic elicitation research identifying unmet patient needs for comfort, guidance, and empathy during routine oral interventions.^8^ This finding was also supported by Rose and colleagues, who described ICU nurses’ recognition of patient behaviors such as the inability to follow commands, gagging/coughing, and pulling at oral tubes as indicative of pain less than 50% of the time.^33^ Recent research demonstrates that lack of pain appraisal or treatment before oral interventions independently predicts responsive patient behaviors (e.g., grimacing, biting, turning the head side to side, gagging). Our study participants recalled similar responsive behaviors.^34^ Assuming agitation rather than pain may result in physical restraint, which contributes to delirium, prolonged ICU treatment, and posttraumatic stress disorder.^35^ Structured appraisal and management of pain, in contrast, is demonstrated to reduce the duration of mechanical ventilation,^2^ nosocomial infection,^36^ and development of posttraumatic stress disorder.^37^

Common characteristics of ICU patients may place them at risk of procedural oral pain, including age 60 years or older, low socioeconomic status, care dependency, comorbidity, and polypharmacy.^38^ Public health studies addressing the needs of older adults demonstrate a link between poor general health and higher levels of painful dental disease.^39^ Canadian data also suggest that those experiencing difficulty accessing dental care due to financial limitations have more prevalent and severe disease.^40^ Demographic trends indicate that future ICU cohorts are more likely than previous cohorts to be older and retain their natural teeth, thereby increasing the likelihood of admission with untreated dental conditions.^39,41^ Participant reports in this study of loose teeth and pain following ICU discharge may signify new or worsening inflammation and infection of the gums. Prior investigation suggests that oral care is a low priority in the ICU, which may explain limited participant recollection of toothbrushing and treatment of oral dryness.^42,43^

Proactive management of procedural pain may improve the patient experience, facilitate successful delivery of preventative oral care, and reduce poor short- and long-term outcomes associated with pain.^9^ Recent innovations in pain include validation of the Critical Care Pain Observation Tool for the detection of mouth and throat pain during routine oral procedures in intubated and tracheostomized adults.^44^ During Critical Care Pain Observation Tool validation, pain was present for 43% of patients during oral suctioning, for 39% during oral swabbing, and for 30% during toothbrushing. Burning sensation and erosive lesions associated with some antimicrobial rinses may also require pain management.^45^ The introduction of a valid pain observational tool for use in nonverbal ICU patients is noted to increase pain assessments and appropriate use of analgesia in medical and surgical ICU patients.^46^

To advance clinical awareness, ICU guidelines should identify oral pain as a target for practice improvement.^7^ According to the World Health Organization, Oral health is essential to general health and quality of life. It is a state of being free from mouth and facial pain, oral and throat cancer, oral infection and sores, periodontal (gum) disease, tooth decay, tooth loss, and other diseases.^47^ Patient satisfaction with pain management is an important benchmark for assessing quality of care.^48^ Moreover, unmanaged oral pain may contribute to agitation and confusion, which may intensify clinical workload.^49^ Clinical practice guidelines can assist clinicians in identifying appropriate care for oral pain, thereby potentiating improved care processes and outcomes.^50^ Future ICU research may explore the impact of procedural oral pain assessment and management on oral care delivery and patient experiences.

A key strength of this study is the use of qualitative interviews paired with object elicitation. We found the presentation of oral care tools to be productive in facilitating patient recall of oral procedures, sources of comfort and pain, and the meanings that participants attribute to those experiences. Emotional responses to the objects revealed a complex patient relationship to oral interventions and clinicians’ delivery methods. Value was attributed to what Kolcaba and Kolcaba described as “comfort”: the relief, ease, and transcendence derived from some oral interventions (e.g., application of moisture with a sponge swab) and gentle delivery methods.^51^ In contrast, other interventions (e.g., oral suctioning) and mechanistic delivery methods were implicated in pain, fear, and suffering. Object elicitation helped to release unexpected recollections of care omissions (e.g., toothbrushing, anticipatory guidance), which emphasize patient expectancies for greater frequency of oral care and empathic delivery methods.^52^ Other researchers explain that objects may act as a third party in the interview, thereby reducing social diffidence^24^ and perceptions of interrogative interviewing, which can inhibit disclosure.^53^ Overall, we found elicitation to advance understanding of the dehumanizing nature of care when pain is not addressed and the potential humanizing value of oral hygiene during serious illness.^54^

Limitations of our research include a convenience sample recruited from the primary study and a single-center design, which may restrict understanding of pain in different settings and populations. A mean duration of oral intubation of 7 days or more may restrict understanding of pain in patients with a shorter duration of treatment. Other possible sources of oral pain include preexisting periodontal disease and use of chlorhexidine oral rinse in the study unit. Similar to prior reports, patients were weak and uncomfortable immediately following ICU discharge. This may have resulted in less detailed or evocative descriptions of experiences and recommendations.^55^ However, including only those participants able to provide detailed descriptions of their experiences may threaten the trustworthiness of the results.^56^ Other qualitative designs, such as those incorporating longitudinal interviews and observation, may be important for exploring and understanding procedural oral pain.

## Conclusion

In this study, participants recalled pain and emotional distress associated with oral care procedures and identified procedural oral pain as a modifiable outcome during mechanical ventilation. Recommended interventions to manage procedural oral pain included comprehensive oral care to prevent oral health deterioration, anticipatory verbal guidance to support patients during oral procedures, and structured pain assessment. Participants identified frequency of oral care and oral pain management as important targets for practice improvement.
